# Health-related quality of life and utility values associated to hypoglycemia in patients with type 1 diabetes mellitus treated in the Brazilian Public Health System: a multicenter study

**DOI:** 10.1186/s13098-017-0206-4

**Published:** 2017-01-28

**Authors:** Luciana Bahia, Rosane Kupfer, Denise Momesso, Debora A. P. Cabral, Balduino Tschiedel, Marcia Puñales, Suzana Lavigne, Cristina F. S. Façanha, Adriana C. Forti, Angela D. N. Mendes, Bernardo R. Tura

**Affiliations:** 1 Universidade do Estado do Rio de Janeiro-UERJ, Rio de Janeiro, Brazil; 2 Instituto de Diabetes e Endocrinologia Luiz Capriglione-IEDE, Rio de Janeiro, Brazil; 3 Instituto da Criança com Diabetes do Rio Grande do Sul-ICDRS, Rio Grande do Sul, Brazil; 4 Centro de Diabetes e Hipertensão de Fortaleza-CIDH, Fortaleza, Brazil; 5 Instituto Nacional de Cardiologia-INC, Rio de Janeiro, Brazil; 6 Visconde de Pirajá 547/501 Ipanema, Rio de Janeiro, 22410-003 Brazil

**Keywords:** Quality of life, Utility, Hypoglycemia, Diabetes

## Abstract

**Background:**

Hypoglycemia is a critical and limiting factor of a good metabolic control and can adversely affect the quality of life of diabetic patients. The aim of the study was to evaluate the health-related quality of life and calculate utilities values associated with hypoglycemia in patients with type 1 diabetes mellitus (T1DM).

**Methods:**

A multicenter, cross-sectional and observational study with T1DM patients from reference centers of the Brazilian public health system was conducted in three cities. Demographic and clinical data were collected, besides details on the frequency and severity of hypoglycemia. Health-related quality of life was assessed using EQ-5D instrument and utility values generated.

**Results:**

221 patients (107 women, 114 men), aged 29.8 ± 11.6 and disease duration of 14.2 ± 9.1 years were included. Most patients (n = 214, 96.8%) reported at least one symptomatic hypoglycemia in the last three months, 68% (n = 150) reported nocturnal episodes and 34.8% (n = 77) reported severe episodes. High frequency (daily or weekly) was observed in 38.6 and 26% of those reporting nocturnal or severe hypoglycemia, respectively. The median visual analog scale was 70 [60–85] for all patients, with differences between those with and without severe hypoglycemia (70 [60–80] vs 80 [61–90]; p = 0.006) and those with high and low frequency (62.5 [50–72.25] vs 70 [60–80]; p = 0.007). The median utility values was 0.801 [0.756–1.000] for all patients, with difference between those with high and low frequency of severe episodes (0.737 [0.628–1.000] vs 0.801 [0.756–1.000]; p = 0.02).

**Conclusions:**

This study shows the high frequency of hypoglycemia in a sample of T1DM patients treated in three reference centers of the Brazilian public health system and the impact of severe episodes on health-related quality of life. Utility values were generated and can be used in economic analysis for treatments that could decrease hypoglycemia and consequently improve quality of life.

## Background

Hypoglycemia is a common event in the lives of patients with type 1 diabetes mellitus (T1DM) and carries high morbidity. The goal of diabetes treatment is to achieve euglycemia without episodes of hypoglycemia, but the frequent occurrence of hypoglycemia is a critical and limiting factor of a good metabolic control in the short and long-term [1]. The episodes may be asymptomatic and go undetected, quickly leading to alterations of consciousness and coma, or accompanied by extremely uncomfortable and disturbing symptoms associated with decreased quality of life. Often patients with diabetes fear more the severe hypoglycemic episodes than the chronic complications of the disease [2]. Hypoglycemia can have several effects on the health-related quality of life, like the acute unpleasant symptoms of the episode itself, fear of risk situations and future episodes, anxiety and reluctance to implement intensive insulin regimen to better metabolic control [3].

The quality of life and utilities are different concepts, however, are related to each other. The concept of utility originates in economic theory that deals with decision making and seeks to develop a model to represent how individuals make decisions when faced with uncertain conditions of health, based on their individual preferences [4]. Utility values are obtained requesting individuals to make judgments about their health status, changes in health status or outcomes, and aim to summarize a variety of aspects and concepts related to health and quality of life in a unique measure [5].

From utility data, it is possible to calculate quality-adjusted life years (QALY), which is a very appropriate measure for clinical effectiveness in economic analyzes (cost-utility studies) in certain contexts and diseases, such as diabetes. There are several methodological approaches to estimate utilities, for example, by validated questionnaires (EQ-5D, SF-6D and HUI3) or discrete choice experiments. In the present study, we opted for using EuroQol (EQ-5D) [6] that is a generic and multidimensional tool that has already been successfully applied in many studies and in several countries, including Brazil [7]. These results can be used for economic evaluations of interventions (prevention, management or treatment) that can affect the quality of life of patients with diabetes.

The aim of the study was to evaluate the health-related quality of life and calculate utilities values associated with hypoglycemia in patients with type 1 diabetes mellitus treated in reference centers of the Brazilian Public Health System.

## Methods

A multicenter, cross-sectional and observational study that collected data from patients with T1DM on health-related quality of life and hypoglycemia.

The study aimed to recruit 300 patients with T1DM in three public reference centers: Instituto da Criança com Diabetes-ICDRS, Porto Alegre; Instituto de Endocrinologia e Diabetes Luiz Capriglione-IEDE, Rio de Janeiro e Centro Integrado de Diabetes e Hipertensão-CIDH, Fortaleza. Included patients had clinical and laboratory diagnosis of T1DM and more than 18 years. Individuals with any physical or mental conditions that limit the understanding and answering of the questions were excluded.

They were recruited consecutively according to the eligibility criteria and attendance to their medical consultations. Patients answered a structured questionnaire administered by a trained interviewer. The questionnaire included demographic data, information of the disease and its treatment, presence of associated medical conditions, information on intensity and frequency of hypoglycemia and health-related quality of life (EQ-5D and visual analogue scale-VAS).

All subjects were invited to participate and signed a term of consent. The study protocol was submitted to the Research Ethics Committee of each of the executing centers.

### Hypoglycemia

Hypoglycemic events were classified as:

1. Symptomatic hypoglycemia: an event during which typical symptoms of hypoglycemia accompanied or not by a measurement of glucose concentration in plasma ≤70 mg/dl.
2. Severe hypoglycemia: an event that requires assistance from another person to actively administer carbohydrate, glucagon, or other resuscitative actions.
3. Nocturnal hypoglycemia: a symptomatic event (documented or not), severe or not, which occurred after 10 p.m. until dawn.

The frequency of hypoglycemic events was defined as daily, weekly, monthly or at least one episode in the last 3 months. High frequency was considered those individuals with daily or weekly episodes, and low frequency for the others.

### Health-related quality of life and utility values

The EQ-5D is a generic instrument that assesses the quality of life through 5 domains: mobility, self-care, usual activities, pain/discomfort and anxiety/depression. For each item, there is gradation 1, 2 and 3 (no problem, some problems and extreme problems), respectively). Combining the results of these five scores produces 243 different health states.

The utility values vary, usually from 0 (equivalent to death) to 1 (equivalent to perfect health), while negative values (worse than death) can be obtained. The different possible health states are judged from this score from 0 to 1, that is from a conceptual point of view, are valued by reference to death states and perfect health.

For each health state generated by EQ-5D, a value of utility was assigned according to the valuation parameters of health status obtained from the Brazilian validation study [7].

### Statistical analysis

The descriptive analysis of the results is presented in terms of mean or median and standard deviation or variance. We used the Mann–Whitney test for comparisons on VAS and utility values. The Kruskal–Wallis test was used to compare categorical variables.

## Results

We included 221 patients, 107 women and 114 men, mean age 29.8 ± 11.6 years, BMI 23.7 ± 4 kg/m^2^ and disease duration of 14.2 ± 9.1 years.

All patients were using rapid and basal insulins. The basal insulin mean dose was 39.5 ± 15.2 and 17.8 ± 10.4 U/day for rapid insulin. Most of the population (74.8%) used NPH insulin as basal insulin, with 55 patients (25.5%) in use of long-action analogs (47 patients with insulin Glargine and 8 with Detemir). The rapid-acting analogs were used by 63.4% of the population (18 Aspart, 119 Lispro and 2 Glulisine ), the others used regular insulin (Table 1). There were two patients with incomplete data on treatment.

**Table 1:** Treatment according to insulin types

Basal insulins		Rapid insulins	
N = 219		N = 219	
NPH	164 (74.48%)	Regular	80 (36.5%)
Glargine	47 (21.8%)	Aspart	18 (8.2%)
Detemir	8 (3.7%)	Lispro	119 (54.3%)
		Glulisina	2 (0.9%)

Most patients (n = 179, 80.9%) reported that they followed the treatment recommendations partially, 21 patients (9.5%) followed all the treatment recommendations, while 17 patients did not follow any guidance (7.69%). Carbohydrate counting was performed by 83 patients (37.8%).

Regarding chronic complications of diabetes and associated medical conditions, 19.4% reported having nephropathy, 21.7% retinopathy, 21.8% neuropathy, 13.6% were hypertensive, 0.9% reported coronary heart disease and 1.8% reported a previous episode of stroke.

The frequency and severity of hypoglycemia episodes are displayed in Table 2.

**Table 2:** Frequency and severity of hypoglycemia episodes

Frequency (%)	Hypoglycemia^a^	Nocturnal	Severe
	N = 214 (96.8%)	N = 150 (68.1%)	N = 77 (34.8%)
Daily	27 (12.6%)	5 (3.3%)	2 (2.6%)
Weekly	104 (48.6%)	53 (35.3%)	18 (23.4%)
Monthly	67 (31.3%)	56 (37.3%)	19 (24.7%)
Once (last 3 months)	16 (7.5%)	36 (24%)	38 (49.3%)

^a^Any symptomatic hypoglycemia excluding nocturnal or severe episode

**Fig. 1:**
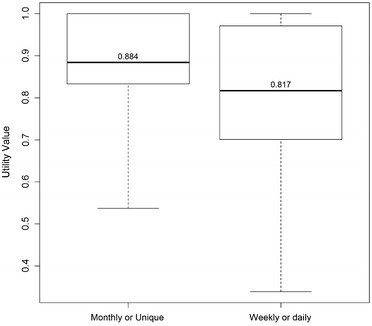
Utility values in patients with severe hypoglycemia (frequent or rare)

**Fig. 2:**
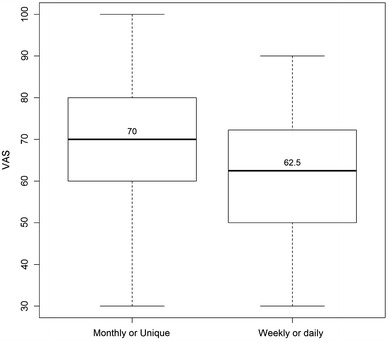
Visual analogue scale in patients with severe hypoglycemia (frequent or rare)

The median value of the visual analogue scale (VAS) was 70 [60–85] and the median utility value was 0.801 [0.756–1.000]. Tables 3 and 4 show the VSA and utility values according to the categories and frequency of hypoglycemia. Figures 1 and 2 demonstrate the utility and VAS according to the frequency of severe hypoglycemia.

**Table 3:** Visual analogue scale and utility values according to the categories of hypoglycemia

Categories	Visual analogue scale				Utility			
	Q1	Median	Q3	p value	Q1	Median	Q3	p value
Hypoglycemia								
No	50	70	85		0.787	0.801	1.000	
Yes	60	70	85	0.39	0.756	0.801	1.000	0.76
Nocturnal hypoglycemia								
No	60	75	85		0.787	0.801	1.000	
Yes	60	70	85	0.27	0.756	0.801	1.000	0.9
Severe hypoglycemia								
No	61	80	90		0.787	0.801	1.000	
Yes	60	70	80	0.006	0.737	0.801	1.000	0.14

**Table 4:** Visual analogue scale and utility values according to the frequency of nocturnal and severe hypoglycemia

Categories	Visual analogue scale				Utility			
	Q1	Median	Q3	p value	Q1	Median	Q3	p value
Nocturnal hypoglycemia								
Absent	60	75	85		0.756	0.801	1.000	
Low^a^	60	70	85		0.787	0.801	1.000	
High^b^	60	70	81.25	0.40^§^	0.737	0.801	1.000	0.74^§^
Severe hypoglycemia								
Absent	61	80	90		0.787	0.801	1.000	
Low^a^	60	70	80		0.756	0.801	1.000	
High^b^	50	62.5	72.25	0.007^§^	0.728	0.737	1.000	0.02^§^

^§^Between low and high frequency of hypoglycemia

^a^Low frequency: one monthly episode or one in the last 3 months

^b^High frequency: daily or weekly episodes

## Discussion

The present study aimed to collect data from a group of patients with T1DM treated in the Brazilian Public Health System (SUS) for generating information on health-related quality of life and utility values associated to hypoglycemia. The study population was derived from reference centers for the treatment of T1DM in the South, Southeast and Northeast regions of Brazil. The treatment with basal-bolus regimen was done by all patients, with 25.5% using slow-acting insulin analogs and 63.4% using rapid-acting analogs. Approximately 20% already had chronic complications of diabetes and only 9.5% followed all the treatment recommendations correctly.

As expected, most of the population reported episodes of hypoglycemia in the last 3 months, with 68 and 34% reporting nocturnal and severe hypoglycemia, respectively. Of the patients who had severe hypoglycemia, requiring third-party help for their recovery, 26% reported high frequency of episodes.

We observed the perception of diabetic patients in relation to their health by Visual Analogue Scale assessment. The result was slightly lower than that found in the Brazilian multicenter study of 3.005 T1DM patients (70 vs 72.5) [8] and much lower than the national average obtained in the sample of 9.148 healthy subjects of similar age (70 vs 82.1) [7]. The comparison of VAS and utility values between groups with and without hypoglycemia did not showed significant difference, but the patients with severe hypoglycemia and those with high frequency of episodes (daily or weekly) had lower values than patients who did not report these episodes. These results are consistent with previous studies that have shown that hypoglycemia negatively influences quality of life in type 2 [9, 10] and type 1 diabetic patients [11].

The evaluation of health-related quality of life has been increasingly recognized as an important area of scientific knowledge, as it takes into account various aspects of the patient’s life, physical and mental well-being, and satisfaction with treatments. Several assessment tools have been proposed to evaluate specifically the diabetic population, but many of them have not yet been validated in Brazil [12]. A limitation of this study was the use of a generic instrument for quality of life assessment, that although often used and previously validated in Brazil, which allows comparison with other populations, is not specific to diabetes and therefore has limitations in reaching some aspects of T1DM patients’ lives.

There are now a substantial number of studies reporting utility scores for people with diabetes and for common complications associated with the disease [13]. Health technology assessment agencies around the world have consistently adopted the inclusion of utilities (or disutilities) associated with health interventions in economic analysis of new technologies. The premise is that possible effects of treatments can add utility decrements or increments and, therefore, should be considered as a parameter for evaluation beyond the efficacy.

Based on the data from the National Euroqol validation study [7], it was possible to obtain, for the first time utility values specifically for the Brazilian population with T1DM and related to hypoglycemia. Although the values were not different between diabetic patients with and without hypoglycemia, possibly by the small sample size, it was observed that patients who had a high frequency of severe hypoglycemias reported a worse perception of their health status. It must be taken into consideration in treatment decisions by prescribers and health policy makers, especially when thinking of populations of children.

## Conclusion

This study shows the high frequency of hypoglycemia in a sample of T1DM patients treated in three reference centers of the Brazilian public health system and the impact of severe episodes on health-related quality of life. Utility values were generated and can be used in economic analysis for treatments that could decrease hypoglycemia and consequently improve quality of life.
